# Haematological findings in 158 cows with acute toxic mastitis with a focus on the leukogram

**DOI:** 10.1186/s13028-021-00576-0

**Published:** 2021-03-12

**Authors:** Ueli Braun, Christian Gerspach, Barbara Riond, Carina Oschlies, Sabrina Corti, Ulrich Bleul

**Affiliations:** 1 Department of Farm Animals, Vetsuisse Faculty, University of Zurich, Zurich, Switzerland; 2 Department of Clinical Diagnostic and Services, Vetsuisse Faculty, University of Zurich, Zurich, Switzerland; 3 Institute of Food Safety and Hygiene, Vetsuisse Faculty, University of Zurich, Zurich, Switzerland

**Keywords:** Cattle, Acute toxic mastitis, Toxaemia, Leukocytes, Leukogram

## Abstract

**Background:**

In cows with acute toxic mastitis (ATM), the leukogram aids in the assessment of the severity of disease. The goal of our study was to compare the leukogram of 158 cows with ATM (cases) and 168 clinically healthy cows (controls). We hypothesised that the leukograms of surviving and non-surviving cows differ and that there are variables of the leukogram with sufficient prognostic potential to be used in the decision to treat or not to treat a cow with ATM. The cows were examined clinically and underwent haematological and biochemical examination of blood and bacteriological culture of milk samples.

**Results:**

All cows with ATM had a poor appetite or anorexia, and 34 cows (21.5%) were recumbent. A single quarter was affected in 119 cows (75.3%), two quarters in 37 cows (23.4%) and three quarters in two cows (1.3%). Bacteriological culture showed Gram-negative pathogens in 100 cows (63.3%), Gram-positive in 15 (9.5%) and yeast in 4 (2.5%). The median total leukocyte count of cases was 4300 cells/µL (interquartile range = 2300–8200/µL), which was significantly lower than 8000 cells/µL (6525–9300/µL) in controls. Except for band neutrophils and metamyelocytes, the counts of all components of the leukogram were lower in cases compared with controls. Significantly more cows with ATM had leukopenia (60.1 vs. 4.1%) or leukocytosis (10.1 vs. 3.0%) than controls. Diseased cows had significantly lower segmented neutrophil counts than controls (860 vs. 2598 cells/µL), and 69.5 and 17.3%, respectively, had counts below the reference interval. Cases had increased band (77.3%) and metamyelocyte (25.0%) counts compared with controls (0.6 and 0%, respectively). In diseased cows, eosinopenia occurred in 66.4% (controls, 1.8%), monocytopenia in 40.6% (4.2%) and lymphopenia in 60.2% (1.8%). Twenty-one diseased cows (16.4%) had a regenerative and 57 (44.5%) had a degenerative left shift. The median neutrophil-to-lymphocyte ratio was 0.97 in diseased cows and 0.63 in controls. Toxic changes in neutrophils including cytoplasmic basophilia and vacuolisation were seen in 101 (91.8%) of 110 blood smears of diseased cows. The leukogram of the surviving and non-surviving cows did not differ significantly, and the hypothesis was rejected.

**Conclusions:**

ATM results in severe changes in the leukogram particularly leukopenia, lymphopenia, and degenerative left shift. The hypothesis that the leukograms of surviving and non-surviving cows differ was rejected. The leukogram has not sufficient prognostic potential to be used in the decision to treat or not to treat a cow with ATM.

## Background

Acute toxic mastitis (ATM) is characterised by typical clinical findings that include listlessness, anorexia, fever or hypothermia and muscle weakness in addition to inflammation of the mammary gland and abnormal mammary secretion. Severely affected cows become dehydrated, recumbent, and develop shock [1]. Changes in the leukogram [2] include leukopenia, neutropenia, degenerative left shift, and neutrophils with toxic changes. The clinical diagnosis of ATM is straightforward and therefore a leukogram is not required for an initial diagnosis. Cows with ATM commonly have leukopenia and neutropenia, which contrasts with other domestic animal species such as the dog in which pronounced neutrophilia with a degenerative left shift occur in response to an inflammatory stimulus. This response typically occurs within six to eight hours after the insult and the degree of response is related to the severity of the inflammation [3]. A thorough knowledge of the process of leukopoiesis aids in the interpretation of changes in the leukogram of cattle with acute inflammation. Leukopoiesis involves the proliferation, maturation, and storage of white blood cells [4, 5]. Pluripotent stem cells differentiate into myeloblasts, which mature through a series of stages that include promyelocytes, myelocytes, metamyelocytes, band cells and mature granulocytes; this so-called proliferative pool accounts for approximately 10–30% and the maturation and storage pools for 65–90% of the granulocytes in the bone marrow [4]. The maturation pool includes metamyelocytes and band neutrophils, which have lost their mitotic potential, and the storage pool comprises band and segmented neutrophils ready to be released into the circulation. The normal transit time for maturation of a myeloblast is about 6–7 days. In several animal species, but not in cattle, the transit time can be reduced to 2–3 days when leukocyte consumption is high. Therefore, the supply of mature leukocytes from the bone marrow rapidly becomes insufficient in cows with high consumption of neutrophils at the site of inflammation. When interpreting a bovine leukogram one must consider that bone marrow response is slower with increased demand for neutrophils [6] and the granulocyte storage pool is smaller compared with other species [7]. This often results in neutropenia, rather than neutrophilia, in the first 24–48 h after the onset of acute inflammation because neutrophils move out of the circulation and into the tissues and the bone marrow cannot meet demand adequately. Approximately 24 h after the start of the inflammatory process, immature leukocytes including band neutrophils, metamyelocytes and occasionally myelocytes are seen in blood smears. The neutrophils often have cytoplasmic basophilia due to staining of certain organelles that are present during early development [8]. Cytoplasmic basophilia is referred to as a “toxic change”, but this is an unfortunate term because the cells have not been damaged by the pathogen and in fact function normally.

The effects of intramammary infection on the total leukocyte and neutrophil counts in cows have been studied experimentally [9–13]. Gram-negative pathogens including *Escherichia coli*, *Klebsiella pneumoniae* and *Serratia marcescens* resulted in neutropenia within 16 h, whereas neutropenia did not occur until 36 h after infection with *Streptococcus uberis* and did not occur with *Staphylococcus aureus* infection. Transient neutropenia was observed 24 h after infection with *Pseudomonas aeruginosa* [12] and from 48 to 168 h period after infection with *Mycoplasma bovis*, which also included lymphopenia from 84 h post infection until the end of the 10-day study [13]. Cows with acute mastitis caused by Gram-negative bacteria had significantly lower leukocyte, segmented neutrophil, monocyte, and lymphocyte counts than cows with mastitis caused by Gram-positive bacteria [14]. Transient neutropenia, often followed by rebound neutrophilia with subsequent normalisation of the neutrophil count, is a good prognostic sign, whereas a continued decrease in neutrophil numbers on day 2 of infection or neutropenia that persists for more than 3–4 days is a poor sign indicating bone marrow suppression [6].

Lymphopenia and monocytopenia are also typical findings in acute mastitis [2]. Stress and exogenous administration of corticosteroids are the most common causes of leukopenia in cattle [6], but infection with viruses, *Anaplasma phagocytophilum*, mycoplasma and other microorganisms as well as septicaemia are other possible causes [15]. Monocytopenia is of lesser significance [16] and cell counts normalise more quickly because the transit time is only 3 days compared with 6 days for neutrophils. Therefore, monitoring monocyte numbers can be useful for predicting the recovery in cases with bone marrow suppression. We hypothesised that the leukograms of cows that either survive or die due to ATM differ and that there are variables of the leukogram with sufficient prognostic potential to be used in the decision to treat or not to treat a cow with ATM.

## Methods

### Animals

One hundred and sixty-eight healthy controls and 158 cows with ATM (cases) were used. The controls were the offspring of cows with bovine spongiform encephalopathy (BSE) and therefore referred by the Federal Veterinary Office Switzerland (now Federal Food Safety and Veterinary Office) to our clinic for examination. The controls were 1–9.6 years of age (mean ± sd = 3.2 ± 1.51 years) and belonged to the Fleckvieh (n = 91), Braunvieh (n = 64), Holstein Friesian (n = 12) and Eringer breeds (n = 1). Clinical findings of the controls were published [17, 18]; all cows had a history of being healthy and the findings of daily clinical examinations during three consecutive days were normal. The 158 cows with ATM included 151 dairy cows and 7 beef cows that were referred to the Clinic for Ruminants, Vetsuisse Faculty, University of Zurich from 2004 to 2016. The dairy cows comprised Swiss Braunvieh (n = 55), Swiss Fleckvieh (n = 50) and Holstein Friesian cows (n = 46), and the beef cows belonged to different breeds or were cross bred. The cows were 2.0–15.1 years of age (mean ± sd = 6.4 ± 2.6 years), and 13 cows were dry. Of the remaining 145 cows, 91 were between 0.5 and 30 days postpartum and 54 were longer than 30 days postpartum or the lactation stage was not known. The duration of disease varied from 0.5 to 30 days (median: 2 days). One hundred and forty-four cows (91%) had received treatment from the herd veterinarian, but only 109 (69%) had been diagnosed as having mastitis.

### Examination of the udder

The examination of the udder was done as described previously [1]. Briefly, the udder, including the teats was inspected, and thoroughly palpated. The mammary secretion was evaluated macroscopically and using the California mastitis test (CMT). Depending on the degree of precipitation or gel formation, the secretion was scored as −, + , +  + or +  +  + . Samples were collected aseptically from untreated quarters with abnormal secretion and a CMT result of +  + or +  +  + and examined bacteriologically using standard methods [1].

### Criteria for the diagnosis of ATM

A diagnosis of ATM was made when cows had acute mastitis accompanied by signs of toxaemia including obtundation, poor skin turgor, enophthalmos, reduced skin surface temperature, prolonged capillary refill time, congested scleral vessels and reduced or absent rumen and intestinal motility. Subjectively, we also considered that cows with toxaemia generally were in worse condition than cows with acute mastitis in the absence of systemic disease and that they failed to respond to routine treatment administered by the primary care veterinarian.

### Haematological, serum biochemical analyses, and bacteriologic examination of milk

The following blood samples were collected from all cattle: 5 mL of EDTA blood for haematological analysis and 10 mL of whole blood for serum biochemistry. Haematological analysis included the determination of haematocrit, total leukocyte count and the concentrations of total protein and fibrinogen. A differential leukocyte count was done in all controls and in 128 cases. The samples of the controls were analysed using the Contraves analyzer AL820 (Contraves, Oerlikon) and those of the cows with ATM with the CELL-DYN 3500 (Abbott Diagnostics Division, Baar). The two analyzers have shown to produce comparable results [19]. The concentrations of serum urea, sodium, potassium, chloride, calcium, inorganic phosphate, and magnesium were determined at 37 °C using an automated analyzer (Cobas-Integra-800-Analyser, Roche Diagnostics, Basel) and the manufacturer’s reagents (Roche-Reagents) according to the International Federation of Clinical Chemistry and Laboratory Medicine (IFCC). Results of the leukocyte count were compared with published reference intervals [20]: Leukocytes (5100–13,300/µL), segmented neutrophils (1700–6000/µL), band neutrophils (0–200/µL), metamyelocytes (0), eosinophils (100–1200/µL), basophils (0–200/µL), monocytes (100–700/µL) and lymphocytes (1800–8100/µL).

Two blood smears were prepared for each cow and stained using a modified Wright’s stain in an automated staining system (HemaTek, Siemens, Switzerland). A manual differential of 100 leukocytes per smear was done by two technicians each with ten years of experience in veterinary haematology. The absolute numbers of lymphocytes, monocytes and granulocytes were determined by multiplying the percentage obtained in the 200-cell differential count with the total leukocyte count from the haematology analyzer. Additionally, the morphology of erythrocytes, leukocytes and platelets was evaluated. The microbiological examination of milk was done as described [1].

### Treatment

The treatment was essentially the same as that used in an earlier study [1]. Ninety-nine cows survived and 59 were euthanased using pentobarbital (Esconarkon, Streuli Pharma, 80 mg/kg BW) administered intravenously.

### Statistical analysis

The program SPSS Version 24 was used for statistical calculations and analysis of the total leukocyte count, segmented, band and metamyelocyte neutrophils, eosinophils, basophils, monocytes, and lymphocytes of both groups. Frequency distributions were determined for both groups and the median and the 5th and 95th percentiles were calculated because the distributions were not normal based on the Shapiro–Wilk test. Differences between medians and frequency distributions of both groups were analysed using ANOVA and the Mann–Whitney U test. To calculate the neutrophil-to-lymphocyte ratio, the segmented, band and metamyelocyte neutrophil counts were divided by the lymphocyte count. The distributions of the ratios were not normal and differences between the medians of both groups were analysed using the Mann–Whitney U test. Pearson correlation coefficients were calculated to assess the relationships between variables.

For the analysis of the leukograms in relation to mastitis pathogens, the latter were divided into 3 groups including Gram-negative (*E. coli*, *Klebsiella* spp., *Proteus* spp.) and Gram-positive bacteria (*S. aureus*, other *Staphylococcus* spp., *S. uberis*, other *Streptococcus* spp., *Trueperella pyogenes*) and yeast. Differences between medians and frequency distributions of the leukograms of the three groups of pathogens were analysed using ANOVA and the Bonferroni post hoc test. Differences between the leukograms of surviving and non-surviving cows were analysed using ANOVA and the Bonferroni post hoc test. Frequency distributions were determined for clinical variables including changes in the udder and the milk of the diseased cows, and the median and the 5th and 95th percentiles were calculated for rectal temperature, heart rate and respiratory rate.

## Results

### Clinical findings

All cows with ATM had poor appetite or anorexia, and 34 cows (21.5%) were recumbent. Many cows had signs of pain including bruxism (n = 29, 18.4%), weight shifting between hind limbs (n = 15, 9.5%), spontaneous grunting (n = 9, 5.7%) and muscle tremors (n = 6, 3.8%). Signs of shock included increased capillary refill time (n = 131, 82.9%), reduced skin turgor (n = 124, 78.5%), sunken eyes (n = 118, 74.7%), cool skin surface temperature (n = 102, 64.6%) and dry muzzle (n = 48, 30.4%). Scleral vessels were congested in 155 cows (98.1%), and the oral mucosa was discoloured in 46 cows (29.3%) (mostly pale and occasionally hyperaemic). The median heart rate was 92 beats per minute (bpm) (5th to 95th percentiles, 68 to 130 bpm), the respiratory rate was 32 breaths per minute (18 to 76 breaths per minute) and the rectal temperature was 38.9 °C (37.5 to 40.4 °C). Rumen motility was reduced in 61 cows (38.6%) and absent in 90 (57.0%); the respective frequencies for intestinal motility were 94 (59.5%) and 25 cows (16.0%). Percussion and simultaneous auscultation and ballottement and simultaneous auscultation were positive in nine cows (5.7%) on the left side and in 53 cows (33.5%) on the right side. Twenty-seven cows (17.1%) had no faeces in the rectum, and in 60 cows (38.0%) the amount was decreased. The latter finding was a subjective assessment when the rectal lumen was incompletely filled with manure or when the examiner’s arm was not immersed in faeces in a manner that occurs in healthy cows. Twenty-four cows had one and two cows had two comorbidities that included endometritis/metritis (n = 6), left displaced abomasum (n = 4), paralytic ileus (n = 3) and pleurisy, aspiration pneumonia, ketosis, abomasal ulcer, caecal dilatation, intertrigo (necrotic dermatitis), gastrocnemius tendon rupture, myopathy and retained placenta in one cow each.

### Udder and milk abnormalities

One hundred and nineteen cows (75.3%) had toxic mastitis in a single quarter, 37 cows (23.4%) in two quarters and two cows (1.3%) in three quarters. The affected quarters were enlarged and firm in 157 (99.4%) cows. In seven cows (4.4%), the skin overlying the quarter was hyperaemic, and in 11 cows (7.0%) the teat was affected by the inflammation. The colour and consistency of the milk were markedly abnormal in 152 (96.2%) and 153 cows (96.8%), respectively, and clots were visible in the secretion of 128 cows (81.0%). A CMT score of 3 (almost a solid gel) was seen in all affected quarters.

Culture of the milk samples showed Gram-negative pathogens (*E. coli*, n = 87; *Klebsiella* spp., n = 12; *Proteus*, n = 1) in 100 cows (63.3%) and Gram-positive pathogens (*S. uberis,* n = 5; *S. aureus*, n = 3; *T. pyogenes,* n = 3; staphylococci other than *S. aureus*, n = 2; streptococci other than *S. uberis, Streptococcus dysgalactiae, or Streptococcus agalactiae*, n = 2) in 15 cows (9.5%). Yeast was recovered in 4 cows (2.5%). Culture yielded no growth in 29 cows (18.4%) that had been pre-treated with antibiotics, and in 10 cows (6.3%) culture results were not available or they were unclear.

### White blood cells, medians, and frequency distributions

**Fig. 1:**
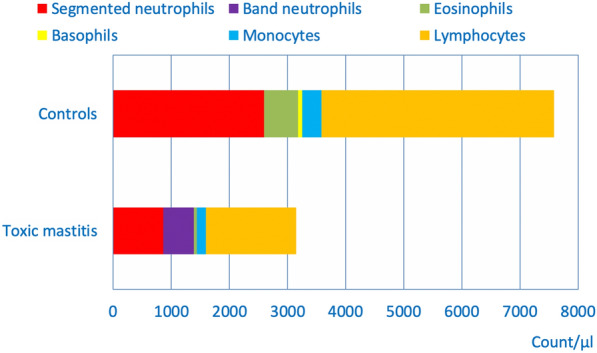
Frequency distributions of the components of the leukogram. Frequency distributions of the components of the leukogram in plasma from 168 healthy control cows and from 158 cows with ATM (medians)

**Fig. 2:**
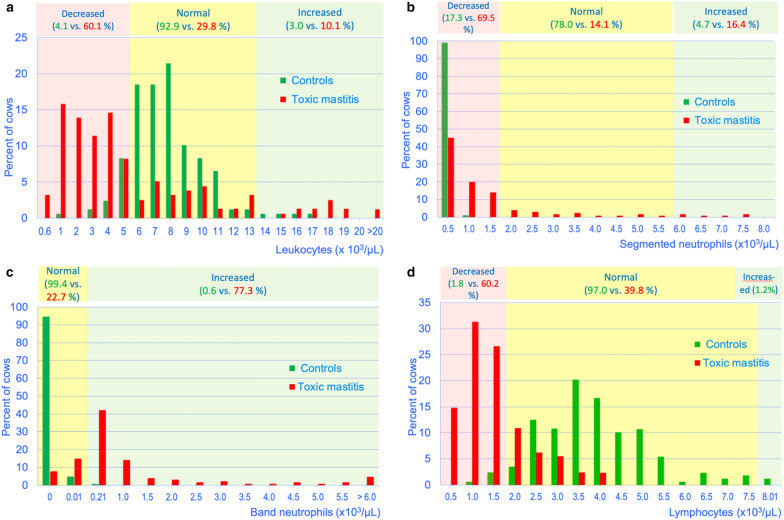
Frequency distributions of the total leukocytes, the segmented neutrophils, the band neutrophils and the lymphocytes. Frequency distributions of the total leukocytes (**a**), the segmented neutrophils (**b**), the band neutrophils (**c**) and the lymphocytes (**d**) in plasma from healthy control cows and from cows with ATM

The leukocyte count was significantly lower in cows with ATM compared with controls (4300 versus 8000 cell/µL; P < 0.01) (Table 1); except for band neutrophils and metamyelocytes, all components of the leukogram were significantly lower in the diseased cows (P < 0.01) (Fig. 1). The frequency distributions also differed between the two groups (P < 0.01) (Fig. 2a); cases had leukopenia (60.1%) or leucocytosis (10.1%) (P < 0.01) significantly more often than controls (4.1%, 3.0%, respectively). The number of segmented neutrophils was significantly lower in 69.5% of cases (860 cells/µL) compared with 17.3% of controls (2598 cells/µL) (P < 0.01) (Fig. 2b). Increased numbers of segmented neutrophils were seen in 16.4% of cases compared with 4.8% of controls. Band neutrophils were seen in 77.3% of diseased cows (median, 530 cells/µL) and in 0.6% of controls (median, 0 cells/µL) (P < 0.01) (Fig. 2c). Metamyelocytes were seen in 25% of cases but did not occur in controls. Eosinopenia was seen in 66.4% of diseased cows and in 1.8% of controls (P < 0.01). Basophils were in the normal range (0 to 200 cells/µL) in all diseased cows and in 97.0% of controls. Monocytopenia (< 100 cells/µL) was seen in 40.6% of cases (155 cells/µL) compared with 4.2% of controls (336 cells/µL) (P < 0.01). Lymphopenia occurred in 60.2% of cases (1550 cells/µL) compared with 1.8% of controls (3998 cells/µL) (P < 0.01) (Fig. 2d).

**Table 1:** Medians and frequency distributions of the components of the leukogram in cows with acute toxic mastitis (ATM) and in clinically healthy controls

Variable	Finding		Controls	ATM
Leukocytes (/µL)	Median		8000 (n = 168)	4300 (n = 158)
	Normal (5100–13,300)^a^ Decreased (600–5099) Increased (13,301–29,100)		92.9% (n = 156) 4.1% (n = 7) 3.0% (n = 5)	29.8% (n = 47) 60.1% (n = 95) 10.1% (n = 16)
Segmented neutrophils (/µL)	Median		2598 (n = 168)	860 (n = 128)
	Normal (1700–6000) Decreased (20–1699) Increased (6001–18,070)		77.9% (n = 131) 17.3% (n = 29) 4.8% (n = 8)	14.1% (n = 18) 69.5% (n = 89) 16.4% (n = 21)
Band neutrophils (/µL)	Median		0 (n = 168)	530 (n = 128)
	Normal (0–200) Increased (201–12,670)		99.4% (n = 167) 0.6% (n = 1)	22.7% (n = 29) 77.3% (n = 99)
Metamyelocytes (/µL)	Median		0 (n = 168)	0 (n = 128)
	Normal (0) Increased (1–13,301)		100% (n = 168) 0%	75% (n = 96) 25% (n = 32)
Eosinophils (/µL)	Median		582 (n = 168)	50 (n = 128)
	Normal (100–1200) Decreased (0–99) Increased (1201–2280)		85.1% (n = 143) 1.8% (n = 3) 13.1% (n = 22)	32.8% (n = 42) 66.4% (n = 85) 0.8% (n = 1)
Basophils (/µL)	Median		70 (n = 168)	0 (n = 128)
	Normal (0–200) Increased (201–339)		97% (n = 163) 3% (n = 5)	100% (n = 128) 0%
Monocytes (/µL)	Median		336 (n = 168)	155 (n = 128)
	Normal (100–700) Decreased (0–99) Increased (701–2170)		94% (n = 158) 4.2% (n = 7) 1.8% (n = 3)	44.6% (n = 57) 40.6% (n = 52) 14.8% (n = 19)
Lymphocytes (/µL)	Median		3998 (n = 168)	1550 (n = 128)
	Normal (1800–8100) Decreased (400–1799) Increased (8101–8721)		97.0% (163) 1.8% (n = 3) 1.2% (n = 2)	39.8% (n = 51) 60.2% (n = 77) 0%

The medians and frequency distributions of all components of the leukogram differ significantly between the two groups (P < 0.01, ANOVA, Mann–Whitney U test)

^a^Reference intervals for all variables: Wood and Quiroz-Rocha[18]

### Frequency distributions of leukocytes relative to the total leukocyte count and left shift

Of the cows with leukopenia, 95.5% had decreased numbers of segmented neutrophils, 72.7% had increased numbers of band neutrophils (Table 2), 67.0% had eosinopenia, 56.8% had monocytopenia and 73.9% had lymphopenia. Of the cows with leukocytosis, 81.2% had increased numbers of segmented neutrophils, 87.5% had increased numbers of band neutrophils and 75.0% had monocytosis, and 56.2% had eosinopenia.

**Table 2:** Frequency distributions of the components of the leukogram in 128 cows with acute toxic mastitis and normal, decreased or increased total leukocyte counts

Variable	Classification	Normal (n = 24) (%)	Decreased (n = 88)^a^ (%)	Increased (n = 16) (%)
Leukocytes				
Segmented	Normal (n = 18)	50.0	4.5	12.5
Neutrophils	Decreased (n = 89)	16.7	95.5	6.3
	Increased (n = 21)	33.3	0	81.2
Band neutrophils	Normal (n = 29)	12.5	27.3	12.5
	Increased (n = 99)	87.5	72.7	87.5
Metamyelocytes	Normal (n = 96)	87.5	71.6	75.0
	Increased (n = 32)	12.5	28.4	25.0
Eosinophils	Normal (n = 42)	29.2	33.0	37.5
	Decreased (n = 85)	70.8	67.0	56.2
	Increased (n = 1)	0	0	6.3
Basophils	Normal (n = 128)	100	100	100
Monocytes	Normal (n = 57)	66.7	42.0	25.0
	Decreased (n = 52)	8.3	56.8	0
	Increased (n = 19)	25.0	1.1	75.0
Lymphocytes	Normal (n = 51)	50.0	26.1	100
	Decreased (n = 77)	50.0	73.9	0

See Table 1 for definitions of normal, decreased and increased ranges

^a^Leukocyte differentials were done in 88 of the 95 cows with leukopenia

All 21 cows with an increased number of segmented neutrophils had a regenerative left shift, i.e., the number of segmented neutrophils exceeded the number of band neutrophils. Fifty-seven cows (44.5%) had a degenerative left shift and thus the number of immature neutrophils (bands and metamyelocytes) exceeded the number of segmented neutrophils.

### Neutrophil-to-lymphocyte ratio

**Fig. 3:**
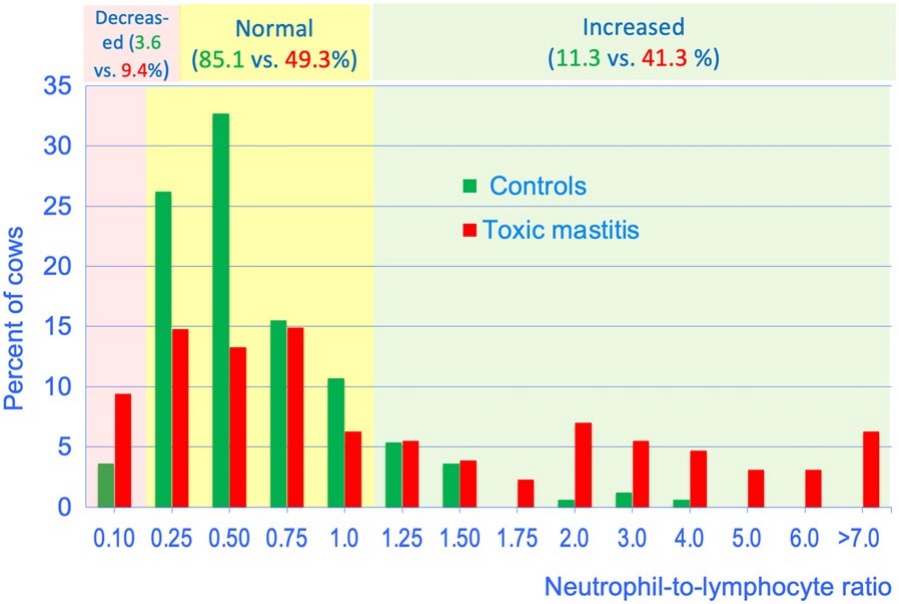
Frequency distributions of the neutrophil-to-lymphocyte ratios. Frequency distributions of the neutrophil-to-lymphocyte ratios in plasma from 168 healthy control cows and from 128 cows with ATM

The median neutrophil-to-lymphocyte (NL) ratio in controls was 0.63 and ranged from 0.28 to 1.53 (5th to 95th percentiles), which was lower than that of diseased cows (0.97, P < 0.01, Mann–Whitney U test). The 5th percentile (0.13) was lower and the 95th percentile was higher (7.46) than in controls. The frequency distributions of the NL ratio differed between the two groups (P < 0.01, chi-square test, Fig. 3).

### Toxic changes in neutrophils

Toxic changes in neutrophils that included cytoplasmic basophilia and vacuolisation were detected in 101 (91.8%) of 110 blood smears. In 58 cows, the occurrence of toxic changes was quantified; 11 cows had changes in 5–10% of neutrophils, 9 had changes in 20–30% and 38 had changes in more than 30% of the neutrophils.

### Effect of Gram-negative and Gram-positive pathogens and yeast on the leukogram

Differences between the medians and frequency distributions of the total leukocyte counts and the numbers of the components of the leukogram associated with Gram-negative (n = 100), Gram-positive (n = 15) and yeast infection (n = 4) were not significant (Table 3), but there were several significant differences between cows of the different pathogen groups and controls. Lymphocyte counts were significantly lower in all three groups (P < 0.01), and cows with Gram-negative infections had significantly lower total leukocyte, segmented neutrophil and monocyte counts and significantly higher band neutrophil counts than controls (P < 0.01). Cows with Gram-positive infections had significantly greater band neutrophil and significantly lower eosinophil, basophil, and monocyte counts (P < 0.01).

**Table 3:** Leukogram of 119 cows with acute toxic mastitis caused by Gram-positive, Gram-negative or mycotic pathogens (medians and 5th to 95th percentiles)

Variable	Controls (n = 168)	Acute toxic mastitis		
		Gram negative (n = 100)	Gram positive (n = 15)	Yeast (n = 4)
Leukocytes (/µL)	8000 (5345–11,910)	3950^a^ (1100–13,570)	5700 (2300–14,880)	3650 (2800–4350)
Segmented neutrophils (/µL)	2598 (1163–5771)	560^a^ (42–9696)	1160 (50–13,142)	1200 (420–2663)
Band neutrophils (/µL)	0 (0–59)	520^a^ (0–5739)	890^a^ (0–4450	135 (40–1133)
Metamyelocytes (/µL)	0 (0)	0^a^ (0–284)	0 (0–112)	0 (0–38)
Eosinophils (/µL)	582 (174–1666)	50^a^ (0–397)	40^a^ (0–270)	125 (110–170)
Basophils (/µL)	70 (0–179)	0^a^ (0–20)	0^a^ (0–36)	0 (0)
Monocytes (/µL)	336 (102–640)	100^a^ (2–1072)	320^a^ (100–1020)	110^a^ (80–145)
Lymphocytes (/µL)	3998 (2292–6959)	1525^a^ (765–3261)	1730^a^ (640–2992)	1545^a^ (840–2028)

^a^Different from control cows P < 0.01

### Effect of duration of disease on the leukogram

There were significant differences in total leukocyte and segmented neutrophil counts among cows with a duration of disease of one, two, three or more than three days (Table 4). Cows examined on the first day of disease had significantly lower total leukocyte (3250 vs. 9200 cells/µL) (P < 0.01) and segmented neutrophil counts (610 vs. 6170 cells/µL, P < 0.05) than cows that had been diseased for more than three days. The counts of other components of the leukogram were not related to duration of disease.

**Table 4:** Leukogram of cows with acute toxic mastitis by duration of illness

Variable	Controls (n = 168)	Duration of illness			
		1 day	2 days	3 days	> 3 days
Leukocytes (/µL)	8000 (5345–11,910)	3250 (n = 70) (900–15,625)	4250 (n = 28) (945–19,920)	5300 (n = 33)^a^ (1500–16,600)	9200 (n = 23)^b,c^ (1560–19,580)
Segmented neutrophils (/µL)	2598 (1163–5771)	610 (n = 63) (50–8492)	480 (n = 23) (24–16,424)	660 (n = 21) (91–9163)	6170 (n = 18)^c,d^ (150–14,522)
Band neutrophils (/µL)	0 (0–59)	460 (n = 63) (0–6948)	490 (n = 23) (0–6924)	1140 (n = 21) (0–6604)	1025 (n = 18) (0–3737)
Metamyelocytes (/µL)	0 (0)	0 (n = 63) (0–180)	0 (n = 23) (0–762)	0 (n = 21) (0–318)	0 (n = 18) (0–125)
Eosinophils (/µL)	582 (174–1665)	40 (n = 63) (0–408)	100 (n = 23) (0–1650)	100 (n = 21) (0–469)	50 (n = 18) (0–325)
Basophils (/µL)	70 (0–179)	0 (n = 63) (0–20)	0 (n = 23) (0–42)	0 (n = 21) (0–74)	0 (n = 18) (0–8)
Monocytes (/µL)	336 (102–640)	120 (n = 63) (10–1292)	150 (n = 23) (20–1340)	190 (n = 21) (2–810)	255 (n = 18) (0–1325)
Lymphocytes (/µL)	3998 (2292–6959)	1430 (n = 63) (654–3334)	1690 (n = 23) (770–4438)	1630 (n = 21) (977–3430)	1820 (n = 18) (840–3164)

^a^Different from day 1 P < 0.05, Kruskal–Wallis-test

^b^Different from day 1 P < 0.01, Kruskal–Wallis-test

^c^Different from day 2 P < 0.05, Kruskal–Wallis-test

^d^Different from day 3 P < 0.05, Kruskal–Wallis-test

### Other laboratory variables

Cows with ATM had significantly higher concentrations of fibrinogen (6.0 vs. 5.0 g/L) in plasma and urea (7.9 vs. 4.5 mmol/L) in serum (P < 0.01) and a significantly lower total erythrocyte count (6.5 vs. 7.3 × 10^6^ cells/µL) as well as lower concentrations of total protein (74 vs. 79 g/L) in plasma and potassium (3.7 vs. 4.2 mmol/L) in serum than controls (P < 0.01).

### Laboratory variables and comorbidities

Apart from the monocyte count, the laboratory results of the 132 cows without comorbidities and the 26 cows with one or two comorbidities did not differ significantly. The median monocyte count of cows without comorbidities was 130 cells/µL (interquartile = 40–410/µL) compared with 320 cells/µL (140–585/µL) in cows with one or two comorbidities (Mann–Whitney test, P < 0.05).

### Laboratory findings and survival

The leukograms of the 99 surviving and 59 non-surviving cows did not differ significantly, but they differed significantly with respect to haematocrit (medians, 33 vs. 36%), red blood cell count (6.39 vs. 6.85 × 10^6^/µL) and the concentrations of total protein (78 vs. 68 g/L), urea (5.7 vs. 8.5 mmol/L) and inorganic phosphorus (1.48 vs. 2.10 mmol/L, all variables P < 0.01).

## Discussion

This study has shown that the total leukocyte count in cows with ATM is considerably lower than in healthy cows. Even though the cows with ATM were on average twice as old as control cows (6.4 vs. 3.2 years), we do not believe that this had an effect on the results. Leukocyte counts of adult cows change little as the cows grow older; Jersey cows aged 3–4 years had a mean white blood cell count of 7.7 ± 1.9 cells and cows of the same breed aged greater than 6 years had a mean count of 7.7 ± 2.5 × 10^3^ cells/µL blood [20]. Likewise, the segmented neutrophil and lymphocyte counts did not differ significantly between the two age groups. Similarly, the fact that the examinations of the control cows and cows with ATM occurred several years apart is unlikely to have affected the results; a fundamental change in leukograms of cattle is not be expected within a few years, and in both studies the blood was analysed within 24 h of collection. Approximately 60.1% of the cows with ATM had leukopenia and only 10.1% had leukocytosis. Segmented neutrophil counts were similar and were decreased in about 69.5% and increased in about 16.4% of the cows. Acute inflammation is generally accompanied by leukocytosis rather than leukopenia, but our findings agree with published reports of clinical [1, 2, 14] as well as experimental acute mastitis [9–13]. The reason for the leukopenia is the low bone marrow reserve of neutrophils in cattle as opposed to other domestic animal species such as the dog [7] and the fact that the transit time from myeloblast to complete maturation is not reduced in the face of high neutrophil demand [6]. In the dog the transit time can be reduced from 6 to 2–3 days with increased demand of segmented neutrophils but not in calves [4] and likely not in adult cattle either. With acute mastitis, large numbers of segmented neutrophils are moved from the circulation into the infected mammary tissue causing leukopenia. This is followed by an increase in leukocyte numbers after about 48 h, often associated with a rebound neutrophilia [6]. For these reasons, the leukocyte count and particularly the segmented neutrophil count were lower in the ATM affected cows that were examined on the first day of disease compared with cows that were referred and sampled at a later stage of disease. Severe leukocytosis with cell counts exceeding 20,000 cells/µL occurred in only two cows (1.3%), which was in agreement with the observation that cattle as well as sheep and goats with acute inflammation have much lower leukocyte peaks than other domestic animal species [15]. A leukocyte count between 20,000 and 30,000 cells/µL is considered extremely high in cattle [15] and only 16.4% of diseased cows had neutrophilia in the present study. Stress is another cause of neutrophilia. Stress leukograms caused by endogenous or exogenous corticosteroids are common in cattle [15] and are characterised by neutrophilia, lymphopenia, eosinopenia and monocytosis [15, 21]. However, a stress leukogram is not associated with a left shift and therefore band neutrophils and metamyelocytes are not seen. In the present study, neutrophilia was always accompanied by a regenerative left shift in affected cows, which means that neutrophilia was due to inflammation and not stress. Degenerative left shift, characterised by an abundance of band neutrophils and metamyelocytes relative to segmented neutrophils, was more common than regenerative left shift and occurred in 44.5% of diseased cows. Degenerative left shift with increased numbers of metamyelocytes is an alarming haematological finding and a poor prognostic sign when it persists for more than 3–4 days [6].

Lymphopenia was detected in 60.4% of all cows with ATM. Lymphopenia in cattle is mostly caused by endogenous or exogenous corticosteroids [6], and in the present study was due to ATM. This was also observed after experimental intramammary infection with *P. aeruginosa* [12] and *M. bovis* [13]. Interestingly, lymphopenia occurred as early as 24 h after *P. aeruginosa* inoculation but only 84 h after *M. bovis* inoculation. Lymphopenia is also seen in viral infections and diseases caused by *Anaplasma* spp. and other bacteria [15].

Metamyelocytes were not detected in blood smears from the controls, which agreed with the veterinary literature [22]. The occurrence of metamyelocytes should therefore be considered pathological and indicative of a massive demand for leukocytes.

In 104 cows (62%), ATM occurred during late pregnancy or during the puerperal period, which most likely was related to impaired neutrophil function in the periparturient period [23, 24] described previously [25, 26]. It is believed that this is related to increased stress and thus increased glucocorticoid concentrations at this lactation stage [27]. Furthermore, recruitment of neutrophils into the mammary gland and neutrophil function are altered in the periparturient period, which may also contribute to the occurrence of mastitis in early-lactation cows [23, 25, 28]. This is supported by the observation that experimental intramammary infusion of endotoxin results in more severe clinical signs in early lactation compared with late lactation in cows [29].

In contrast to other domestic animal species, cows have more lymphocytes than neutrophils in the circulation and therefore a relatively low NL ratio of about 0.5 [7, 15]; in the present study, the controls had a median NL ratio of 0.63. The significantly larger NL ratio of 0.97 in the cows with ATM was due primarily to lymphopenia (in 60.2%) rather than neutrophilia (only 16.4%). Similarly, cows with sole ulcer had a NL ratio of 1.04, which was significantly greater than that of healthy cows even though neutrophil and lymphocyte counts did not differ significantly between the groups [30]. It was therefore recommended to include the NL ratio in the assessment of a blood cell count to emphasise the difference in absolute neutrophil and lymphocyte numbers [6].

Cytoplasmic basophilia combined with vacuolization or vacuolation in all but 8.2% of the examined blood smears was indicative of toxic change [6]. This confirms that the cows with acute mastitis had toxaemia because the changes are generated when maturation of neutrophils in the bone marrow is hindered because of acute and severe inflammation [15].

Cows with ATM caused by Gram-negative and Gram-positive bacteria did not differ significantly with respect to total leukocyte, segmented and band neutrophil, monocyte, and lymphocyte counts. Differences in cell counts between cows with Gram-negative and Gram-positive infections were significant in an earlier study but the number of cows was larger in that study [14]. The observation that cows with Gram-positive infection tended to have lower total leukocyte and segmented neutrophil counts and significantly lower eosinophil, basophil, monocyte, and lymphocyte counts than controls indicates that ATM caused by Gram-positive pathogens constitutes a severe strain on the bone marrow. It should be remembered that differentiation of mastitis caused by Gram-negative and Gram-positive pathogens requires bacteriological examination and is not possible based on clinical signs alone [2].

Increased haematocrit and azotaemia in cows with ATM was likely attributable to prerenal causes. The increased fibrinogen concentration points to the role of fibrinogen as a positive acute-phase protein [20, 31]. Like haptoglobin, it increases rapidly in response to inflammation and was therefore higher in cows that were diseased longer than three days compared with cows on the first day of disease. In cattle, hyperfibrinogenaemia is considered as good as or better than the neutrophil count for determining inflammation [20]. Generally, the degree of increase in concentration of positive acute-phase proteins parallels the severity of inflammation. Fibrinogen is preferred by many because it is easy to measure.

The hypothesis that the leukograms of surviving and non-surviving cows differ was rejected. The two groups differed significantly with respect to other laboratory variables (haematocrit, red blood cell count, concentrations of total protein, urea, and inorganic phosphorus); however, the prognostic value of individual laboratory variable was limited because of considerable overlap of the values between the two groups.

We assume that paralytic ileus, left displaced abomasum, abomasal ulcers, caecal dilatation, ketosis, rupture of the gastrocnemius tendon and myopathy were sequels to ATM. Aspiration pneumonia most likely resulted from an iatrogenic event at the home farm. Other comorbidities (metritis/endometritis, retained placenta, intertrigo) were not related to toxic mastitis.

We were surprised that only about 69% of all ATM cases were diagnosed by the primary care veterinarian. This may have been due to other clinical signs, such as poor general health status, poor rumen motility, low faecal output and positive percussion and/or ballottement and simultaneous auscultation, which were considered diagnostic of other disorders. This emphasises the importance of a comprehensive clinical examination that includes the assessment of the udder and the milk.

## Conclusions

ATM is accompanied by severe changes in the leukogram, chiefly leukopenia, and degenerative left shift. The leukogram aids in a better understanding of the disease. Leukograms like those presented in the present study strongly suggest ATM or toxaemia of another aetiology.

## Data Availability

The datasets used and analysed for this study are available from the corresponding author on reasonable request.
